# One-Step Hydrothermal Fabrication of Three-dimensional MoS_2_ Nanoflower using Polypyrrole as Template for Efficient Hydrogen Evolution Reaction

**DOI:** 10.1038/srep42309

**Published:** 2017-02-14

**Authors:** Xin Lu, Yingwu Lin, Haifeng Dong, Wenhao Dai, Xin Chen, Xuanhui Qu, Xueji Zhang

**Affiliations:** 1 Institute for Advanced Materials and Technology, University of Science & Technology Beijing, Beijing 100083, P.R. China; 2 Beijing Key Laboratory for Bioengineering and Sensing Technology, Research Center for Bioengineering and Sensing Technology, School of Chemistry & Biological Engineering, University of Science & Technology Beijing, Beijing 100083, P.R. China

## Abstract

Herein, a facile and cost-effective strategy for hydrothermal synthesis of three-dimensional (3D) MoS_2_ with adequate active edge sites and advanced hydrogen evolution reaction (HER) performance using polypyrrole (PPy) as template is reported. The MoS_2_ is first thermally nucleated using hexaammonium heptamolybdate tetrahydrate (NH_4_)_6_Mo_7_O_24_·4H_2_O and thiourea as precursor in the presence of PPy, and then they are further annealed to remove PPy at higher temperature to generate 3D MoS_2_-P. Morphology and composition characterizations reveal that the 3D MoS_2_-P exhibits a nanoflower morphology. It presents larger stretched “thin folding leaves” and higher mesoporous volume of 0.608 cm^3^ g^−1^ than the MoS_2_ without PPy as template. Importantly, the 3D MoS_2_-P shows enhanced HER catalytic activity (onset potential at −100 mV) than previously reports that MoS_2_-based HER catalysts. The large “thin folding leaves” possessing efficient edge active sites and defects are responsible to for the enhanced HER performance, while the high mesoporous volume facilitates the transfer of reaction substrate. Our study provides a facile and cost-effective method for synthesis of 3D MoS_2_ with advanced HER performances, which has great potential for larger-scale production and practical industrial applications.

Hydrogen is a green and sustainable energy, acting as attractive alternative of traditional fossil fuels to alleviate the energy crisis and environmental pollution^1^,^2^. Electrolytic water is an efficient and clean technology for hydrogen evolution^3^, and Pt-based noble metal electrocatalysts are commonly employed in hydrogen evolution reaction (HER) to improve the reaction efficiency. However, the high cost and rare reserve are still hinder its practical application^4^. Efficient alternatives are under urgent need. Recently, two dimensional layered molybdenum disulfide (MoS_2_) based materials are emerging as the promising HER. It was supported by both theoretical calculations and experimental studies^5^,^6^. Density function theory (DFT) calculation revealed that the thermodynamic free energy of H adsorption on the unsaturated sulfur atoms at MoS_2_ edge sites was fit for HER application^7^. Additionally, its relatively low cost, abundant reserve and good stability make MoS_2_ a promising alternative candidate of Pt in HER^8^,^9^. However, the relatively lower catalytic activity of bulk MoS_2_ compared to Pt trigger intense research that improve the catalytic capability^10^.

Generally, three strategies are employed to improve the catalytic properties of MoS_2_. First, much effort has devoted to generation of defects to obtain more active edges per unit area, and thus more active sites. Advanced hydrothermal synthesis methods^11^, including plasma-engineered^12^,^13^ or rough substrate chemical vapor deposition (CVD)^14^ are designed to generate defects for enhanced HER. Xie *et al*. developed a hydrothermal routine and paved the way of engineering defects into MoS_2_ using high concentration of the precursors and different amounts of thiourea^11^. The second strategy strives to strengthen the electrochemical properties by atomic-scale modification including chemical doping^15^, adjustment of the metastable 1T-phase^16^,^17^ and strain treating^18^. Furthermore, fabrication of efficient 3D MoS_2_ such as porous nanosheets^19^, vertical nanoflakes^20^, core-shell MoO_3_-MoS_2_ nanowires^21^,^22^ and double-gyroid morphology^23^ etc. are explored to increase specific surface area and active sites, leading to high catalytic activity. Zhang *et al*. designed an edge-rich and highly ordered MoS_2_ naonosheets rooting into polyaniline nanofibers, It showed good catalytic properties and stability^21^. Kibsgaard *et al*. engineered the surface structure of MoS_2_ to preferentially expose edge sites for improved catalytic activity by successfully synthesizing contiguous large-area thin films of a highly ordered double-gyroid MoS_2_ with nanopores^23^. Our group designed a 3D nitrogen-doped graphene supported MoS_2_ as an advanced HER catalyst^24^. Among them, various 3D MoS_2_ structures with high-surface and exposed active sites present great prospect in large-scale and practical applications.

Although reasonable progresses have been achieved in fabrication of 3D structure, it still suffers from some deficiencies such as complicated operation with multiple steps and inefficient catalytic activity. It is highly desirable to develop a facile and straightforward approach to fabricate cost-effective MoS_2_-based catalysts with high HER activity.

Herein, a one-step hydrothermal synthesis route for 3D MoS_2_ flower with advanced HER performance using PPy (MoS_2_-P) was developed. The PPy shows excellent conductivity. Specially, it displays good stability^24^ to keep its morphology during the hydrothermal synthesis, which is good candidate for soft template. Moreover, it has been demonstrated that the molybdenum sulfide anions prefer to attach and dope into PPy, inducing formation of MoS_2_ nanosheets on the surface of the PPy instead of forming independent nanoflowers^25^. Comprehensive characterizations revealed a nanoflower morphology of the resulting MoS_2_-P. It presented larger area “thin folding leaves” and much more mesoporous pore than the counterpart without PPy as template. Enhanced HER performance was demonstrated. The “thin folding leaves” possessed adequate edge active sites, enabling the enhanced HER performance. The higher mesoporous volume facilitates efficient transfer of reaction substrate. The PPy template could generate nanoflower morphology with larger stretched “thin folding leaves” and higher mesoporous volume as well as much more tortuous and cleaved lattice structures than the MoS_2_ without PPy as template. By adjusting the concentration of the PPy, both the morphology and the defects can be controllably engineered. The proposed method is a facile and cost-effective, providing great potential for larger-scale production of 3D MoS_2_ with advanced HER performances and practical industrial application.

## Results

**Figure 1:**
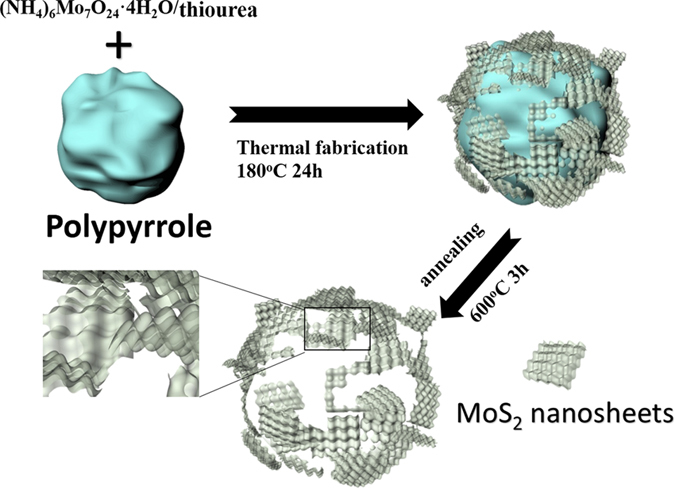
Schematic illustration of the morphological evolution process of the MoS_2_-P.

The one-step hydrothermal synthesis method is schematically illustrated in Fig. 1. (NH_4_)_6_Mo_7_O_24_·4H_2_O and thiourea were chosen as the precursor for large-quantity of MoS_2_ preparation in the presence of PPy, The PPy was employed as the template to generate a MoS_2_ structure with large surface area and adequate active edges. The (NH_4_)_6_Mo_7_O_24_·4H_2_O, thiourea and PPy were simultaneously transferred into the Teflon-lined stainless-steel autoclave for hydrothermal synthesis to generate MoS_2_/PPy composite. The resulting composite was further annealed at higher temperature to remove the PPy^24^,^26^ to produce 3D MoS_2_-P. The PPy played critical roles in the formation of unique morphology and electrochemical performance of the MoS_2_-P, which will be addressed in the following section.

**Figure 2:**
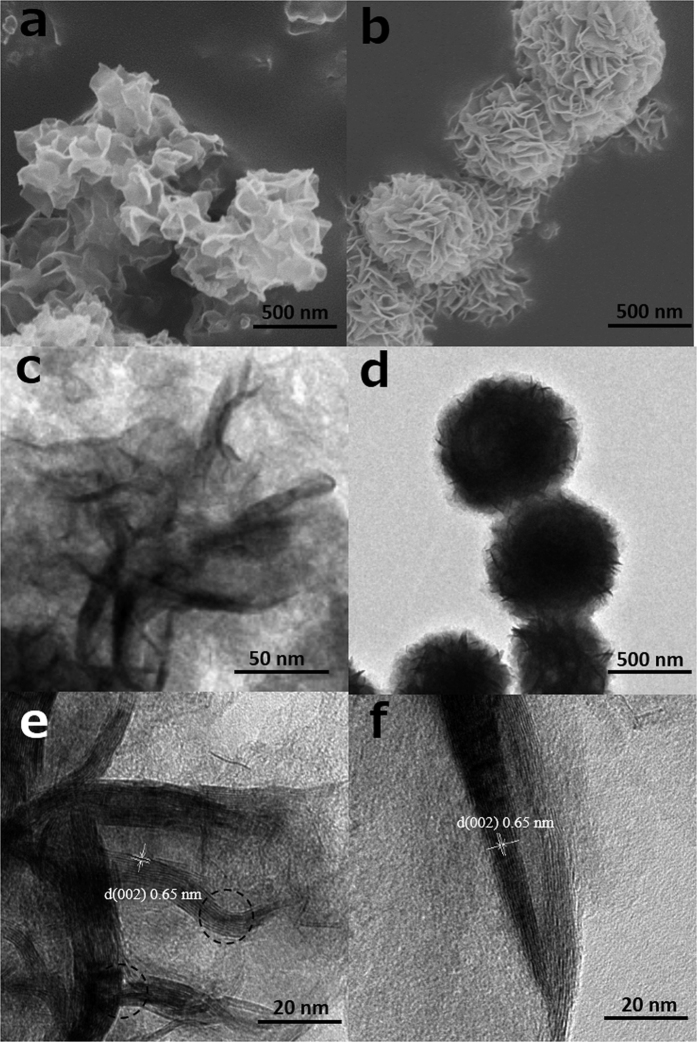
(**a**) SEM image of MoS_2_-P; (**b**) SEM image of MoS_2_; (**c**) TEM image of MoS_2_-P; (**d**) TEM image of MoS_2_; (**e**) HRTEM image of MoS_2_-P; (**f**) HRTEM image of MoS_2_.

Scanning electron microscope (SEM) and transmission electron microscope (TEM) were employed to characterize the morphology of the synthesized composites. As shown in Fig. 2, in comparison with the MoS_2_ without PPy as template displaying a compact nanoflower structure (Fig. 2b), the MoS_2_-P presented a nanoflower morphology with larger stretched “thin folding leaves” (Fig. 2a). PPy acts as a soft template for MoS_2_ in the experiments, providing a substrate to support the growth of MoS_2_. Thus, the morphology of the PPy will influence of the morphology of MoS_2_-P. The SEM image (Fig. S1) shows that the morphology of PPy is an irregular sphere with rough surface, which provide a template for the formation of nanoflower of MoS_2_-P (Fig. 2b). As shown in Fig. S2, the annealing process could effectively remove the PPy^24^. The annealing process also induce tortuous and cleaved lattice in the MoS_2_-P. The large “thin folding leaves” of the nanoflower structure possessing efficient edge active sites and defects are responsible to the enhanced HER performance. The decrease of carbon component during the annealing process further confirmed the effective removal of PPy (Fig. S3). TEM image confirms the larger stretched thin films of MoS_2_-P (Fig. 2c) compared to the closed nanoflower of MoS_2_ without PPy as template (Fig. 2d). These results revealed that the PPy template effectively inhibited the aggregation of MoS_2_ during hydrothermal synthesis process. The formation of stretched “thin folding leaves” is vital for HER, because the adequate exposure of active sites facilitate the efficient substrate accessibility compared to the MoS_2_ that with lots of active edges hided inside the nanoflowers. The high-resoflution TEM (HRTEM) image analysis of MoS_2_-P displayed a clean lattice structure with an interplanar spacing of 0.65 nm (Fig. 2e) ascribed to the (002) planes of MoS_2_^11^, similar to the MoS_2_ (Fig. 2f), which suggested that the MoS_2_ was successfully synthesized. Importantly, numerous tortuous and cleaved lattice structures were observed in the MoS_2_-P (circle in Fig. 2e), indicating the formation of numerous defects during the PPy-assisted MoS_2_ hydrothermal synthesis.

**Figure 3:**
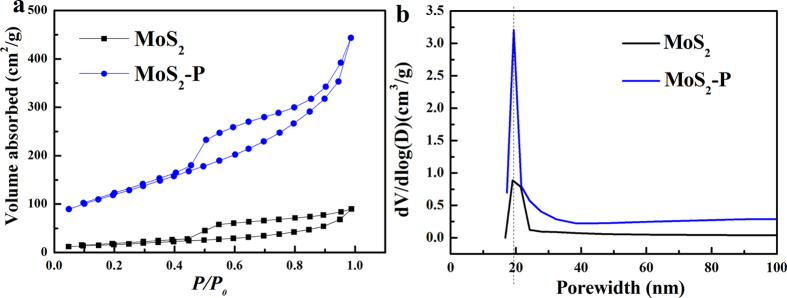
(**a**) Nitrogen adsorption-desorption characterizations of MoS_2_-P (blue curve) and MoS_2_ (black curve); (**b**) the pore size distribution of MoS_2_-P (blue curve) and MoS_2_ (black curve).

As shown in Fig. 3a, both of the nitrogen (N_2_) adsorption-desorption isotherms of the MoS_2_ and MoS_2_-P presented typical IV isothermals hysteresis loop associated with large size mesoporous. The H3 hysteresis loop of MoS_2_-P and MoS_2_ indicated the presence of slit nanopore^27^. Brunauer–Emmett–Teller (BET) calculation revealed that MoS_2_-P displayed six times larger surface area of 431.2 m^2^ g^−1^ than that of MoS_2_ (60.3 m^2^ g^−1^). Although MoS_2_-P and MoS_2_ displayed similar pore size of 19 nm, MoS_2_-P showed a four-fold higher pore volume of 0.608 cm^3^ g^−1^ than that of MoS_2_ with a pore volume of 0.156 cm^3^ g^−1^ (Fig. 3b). Thus, the PPy and annealing process rendered MoS_2_-P with large surface area and adequate nanopores.

**Figure 4:**
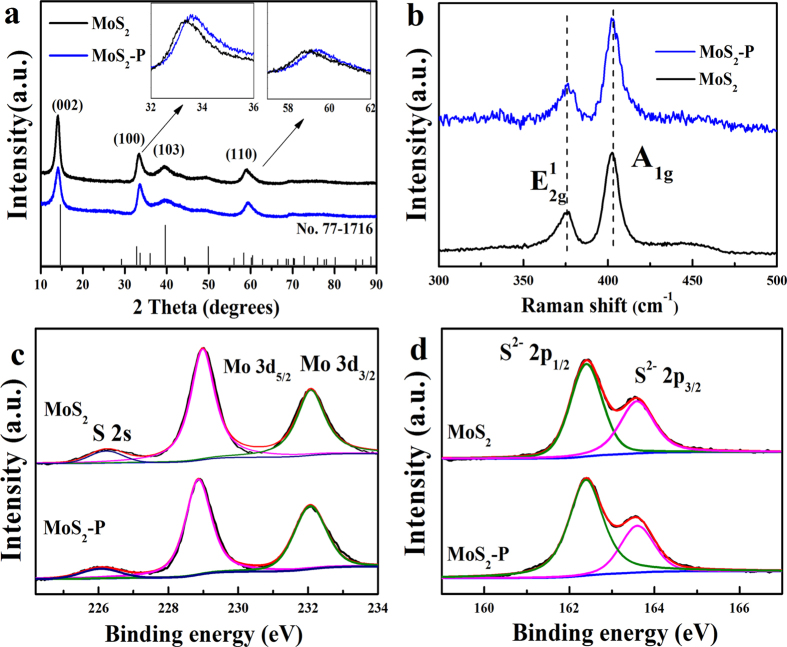
Characterization of MoS_2_ and MoS_2_-P by (**a**) XRD; (**b**) Raman spectroscopy and High-resolution (**c**) Mo 3d and (**d**) S 2p XPS spectra. Inset a: the magnified image of the (100) and (110) peak of the MoS_2_ and MoS_2_-P.

As shown in Fig. 4a, in agreement with MoS_2_ (black curve), the characteristic peaks of MoS_2_-P at 2θ = 14.3, 33.6, 29.7, 59.0 corresponding to the (002), (100), (103), (110) planes (blue curve). It confirmed the well defined and hexagonally symmetric structured of MoS_2_ (JCPDS card no. 77–1716). It indicated less layers of MoS_2_-P than MoS_2_ according to Scherrer analysis of the half maximum (FWHM) value in the (002) diffraction peak^17^ (Fig. 4a). The slight shift of (100) and (110) between MoS_2_ and MoS_2_-P was resulted from the crystal lattice tortuosity (λ = 2dsinθ) (Fig. 4a, inset). The recovery of characteristic peaks in MoS_2_-P compared to PPy encapsulated MoS_2_ (PPy-MoS_2_) (Fig. S4) as well as the thermogravimetric analysis curve of MoS_2_-P (Fig. S5) proved the annealing process removed effectively the PPy. The two strong characteristic peaks located at 376 and 402 cm^−1^ were ascribed to E_2g_, A_1g_ respectively. It suggested the resulted MoS_2_-P (Fig. 4b, blue curve) and MoS_2_ (Fig. 4b, black curve) were mainly 2H-MoS_2,_ and the slight red shift of A_1g_ in MoS_2_-P compared to MoS_2_ was caused by the crystal lattice tortuosity^28^.

The components of the MoS_2_ samples were investigated by X-ray photoelectron spectroscopy (XPS). The MoS_2_ and MoS_2_-P showed the characteristic peaks of the Mo 3d, S 2p and O 1s (Fig. S6). The appearance of weak N 1s peak located at 396 eV and slight increase of C1s at 285.2eV in MoS_2_-P (Fig. S6b) compared to MoS_2_ (Fig. S6a) was resulted from carbonization remnants of PPy during the annealing process. It suggested that PPy might carbonized partially rather than decomposed completely, and it might also generated active nitrogen hybrid species for enhanced HER performance^24^. As shown in the Mo 3d spectrum of MoS_2_-P (Fig. 4c, blue curve), the peak located at 229.8 and 232.9 eV is assigned to Mo 3d_5/2_ and Mo 3d_3/2,_ respectively^29^,^30^, which further suggested the resulting MoS_2_-P was mainly consisted of 2H semiconducting structure^16^ (Fig. 4c, black curve). Similarly, the characteristic peaks of S 2p spectrum in MoS_2_-P (Fig. 4d, blue curve) at 162.4 and 163.5 eV attributed to S 2p_3/2_ and S 2p_1/2_ were presented (black curve), indicating the domain oxidation state of S^2−^ 31. The XPS analysis revealed that the MoS_2_-P and MoS_2_ have little difference in elemental composition and bonding configuration. Similar to MoS_2_, the MoS_2_-P displayed a strong absorption 671 nm, and the band gap was calculated to be 1.52 eV (Fig. S6), agreeing with previous report^32^.

**Figure 5:**
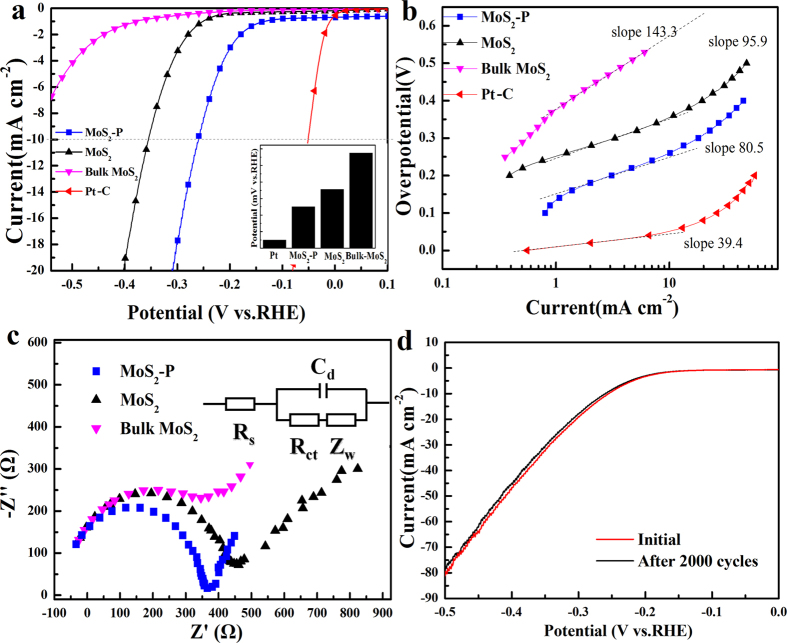
(**a**) Polarization curves obtained of catalysts as indicated; inset: the overpotential of catalysts when the current is 10 mA cm^−2^ (**b**) Corresponding Tafel plots recorded on glassy carbon electrodes with a catalyst loading of 0.28 mg cm^−2^. (**c**) Nyquist plots of the MoS_2_ catalyst recorded. Inset c: equivalent circuit of the EIS spectroscopy. (**d**) Durability test showing negligible current loss even after 2000 cycles.

The HER electrochemical performance of the MoS_2_-P was further investigated. Figure 5a shows representative linear sweep voltammetry (LSV) response for the bulk MoS_2_, commercial Pt-C, MoS_2_ and MoS_2_-P. The MoS_2_ exhibited superior HER performance (black curve) than the raw bulk MoS_2_ (purple curve)^11^. The MoS_2_-P (blue curve) displayed enhanced HER catalytic activity with negative onset overpotential of 100 mV than that of MoS_2_ with an onset overpotential of 170 mV. The MoS_2_-P presented superior an overpotential of −251 mV for 10 mA cm^−2^ to the MoS_2_ of 350 mV and bulk MoS_2_ of 578mV (Fig. 5a, inset). The MoS_2_-P displayed a smaller Tafel slope of 80.5 mV/dec (Fig. 5b, blue curve) than MoS_2_ of 95.9 mV/dec (Fig. 5b, black curve) and bulk MoS_2_ of 143.3 mV/dec (Fig. 5b, purple curve). These results confirmed the enhanced HER performance of MoS_2_-P than MoS_2_ and bulk MoS_2_.

To obtain more information about the intrinsic catalytic activity, the turnover frequency (TOF) for the active sites of different MoS_2_ catalysts was calculated using the roughness factor method according to the following equations^33^.


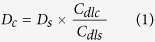



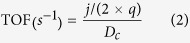


where *D*_*c*_ and *D*_*s*_ were the density of active sites for catalyst (Sites/cm^2^) and standard sample (Sites/cm^2^), the *C*_*dls*_and *C*_*dlc*_ were the double layer capacitor (*C*_*dl*_) for standard MoS_2_ (60 μF cm^−1^) and catalysts calculated by the cyclic voltammetry (CV) experiment at different scan rates (Fig. S7), the *j* (A cm^−2^) was the current density of LSV at −400 mV and *q* was the elementary charge (1.6 × 10^–19^ C). The active sites density of MoS_2_-P was 3.35 × 10^17^ sites/cm^2^, 1.2 times and 27 times higher than that of MoS_2_ (2.85 × 10^17^ sites/cm^2^) and that of bulk MoS_2_ (1.24 × 10^16^ sites/cm^2^), respectively. The MoS_2_-P presented enhanced TOF of 0.85s^−1^ to MoS_2_ (0.67s^−1^) and bulk MoS_2_ (0.54s^−1^), further indicating advanced HER catalytic activity of MoS_2_-P. Electrochemical impedance spectroscopy (EIS) was used to characterize the interfacial reaction and electron-transfer kinetics in HER. As shown in Fig. 5c, MoS_2_-P displayed the lowest faradaic impedance and smallest charge transfer resistance (R_ct_) among these MoS_2_ catalysts. For the long-time durability, the MoS_2_-P showed a negligible decrease in the current density after a long period of 2000 potential-cycling between 0 and −0.5 V, indicating the outstanding electrochemical stability and its promising potential for the practical application (Fig. 5d).

**Figure 6:**
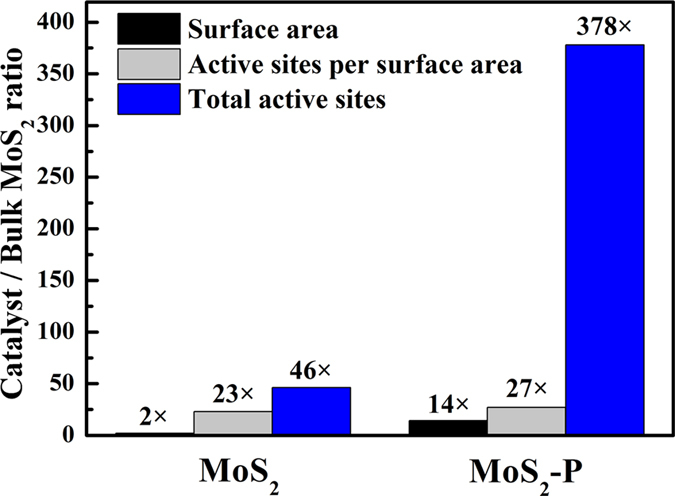
Ratios of surface area, active sites per surface area and total active sites of MoS_2_ and MoS_2_-P versus the bulk MoS_2_.

Briefly, the most important step involved in the HER process is the hydrogen adsorption, which require appropriate Gibbs free energy of the catalyst. Increasing theoretical and experimental reports confirmed the Gibbs free energy of H adsorption on the unsaturated atoms at MoS_2_ edge active site are favorable to hydrogen adsorption, leading to the efficient hydrogen evolution^7^ (Fig. S8). We further compared the HER performance of the MoS_2_-P to previous reports of MoS_2_-based HER catalysts. As shown in Table S1, the MoS_2_-P showed more competitive performance than most of the previous MoS_2_-based catalyst. The enhanced HER performance could be explained as follows: First, the unique nanoflower morphology of large stretched “thin folding leaves” allowed considerable active site exposure for HER (Fig. 2a and c). The quantity of total active sites (calculated by the surface area multiply the density of active sites for catalyst (Sites/cm^2^)) for MoS_2_-P is 8.2 times higher than that of MoS_2_ and 378 times higher than that of bulk MoS_2_ (Fig. 6). Secondly, the numerous defects resulted from the tortuous and cleaved lattice formed during the annealing process also contribute to enhance HER performance (Fig. 2e). Additionally, the high mesoporous volume of MoS_2_-P facilitates the efficient mass transfer.

## Discussion

In this study, we have developed a facile and cost-effective strategy for large-scale synthesis of 3D MoS_2_ nanoflower with large stretched “thin folding leaves” and considerable nanopores by using a PPy-assisted one-step hydrothermal routine. Microscopic and spectroscopic tools including SEM, TEM, HRTEM, BET, XRD, XPS and Raman spectroscopy was employed to comprehensively characterize the morphology and component of the MoS_2_-P. Electrochemical characterizations demonstrated that the prepared MoS_2_-P displayed advanced HER performance. It presenting superior onset overpotential, Tafel plot and lower faradaic impedance than MoS_2_ without using PPy as template, which was competitive to most of the reported analogous MoS_2_-based catalyst. It also displayed long outstanding electrochemical stability for the practical application. It was demonstrated that the high quantity of exposed active sites on the large surface and the defects formed during the hydrothermal synthesis synergistically contribute to the advanced HER catalytic activity, while unique mesoporous structure facilitates the accessibility of the reacted substrate. The facile and cost-effective method for larger-scale synthesis 3D MoS_2_ with advanced HER performances holds great promising in practical industrial application.

## Methods

### Materials and Reagents

(NH_4_)_6_ Mo_7_O_24_ · 4H_2_O, Polypyrrole (PPy, un-doped, extent of labeling: ~20 wt. % loading, composite with carbon black and the CAS number is 30604-81-0) and Pt/C (10% Pt) were obtained from Sigma-Aldrich. Thiourea and KOH were from Sinopharm Chemical Reagent Co., Ltd. Sulfuric acid (H_2_SO_4_, 95–98%) and ethanol (99.9%) was purchased from Beijing Chemical Works. All aqueous solutions were prepared with ultrapure water obtained from a Millipore water purification system (≥18 MΩ, Milli-Q, Millipore).

X-ray diffraction (XRD) was performed by a Rigaku X-ray diffractometer with Cu KR target. The porosity was measured with a nitrogen adsorption-desorption isotherm using a surface area analyzer (QuadraSorb SI 2000–08, Quantachrome Instruments). The morphologies of products were observed under a field-emission scanning electron microscope (SEM; HITACHI S-4800) and a transmission electron microscope (TEM; JEM-2010, 200 kV). X-ray photoelectron spectroscopy (XPS) analysis was performed using an AXIS ULTRADLD instrument equipped with an Al Kα X-ray source. Raman spectra were recorded on an InVia-Reflex Raman microscope with a laser excitation wavelength of 532 nm.

### Materials Preparation of MoS_2_ and MoS_2_-P

MoS_2_-P were synthesized by a hydrothermal synthesis; typically, 44.8 mg polypyrrole (PPy), 123.625 mg hexaammonium heptamolybdate tetrahydrate (0.1 mmol, (NH_4_)_6_ Mo_7_O_24_·4H_2_O, i.e. 0.7 mmol Mo) and 228.375 mg thiourea (3 mmol) were dissolved in ultrapure water (20 mL) under vigorous stirring to form a homogeneous solution. Then, the solution was transferred into a 25 mL Teflon-lined stainless steel autoclave and maintained at 180 °C for 24 h, and the reaction system was then cooled to room temperature. The final product was washed thoroughly with water and ethanol to remove any possible ions, and the as-prepared hydrogel was directly dehydrated via a freeze-drying process to maintain the 3D monolithic architecture, and then annealed at 600 °C for 3 h under argon. As a control, the MoS_2_ was prepared in a similar procedure except using PPy as template.

### Electrochemical Characterization

Electrochemical measurements were performed using a CHI 852C electrochemical workstation (Shanghai Chenhua Instrument Co., China) with a standard three-electrode setup in 0.5 M H_2_SO_4_ aqueous solution. A saturated calomel electrode (Hg/HgCl_2_ in saturated KCl) and a graphite rod were used as the reference electrode and the counter electrode, respectively. A glass carbon rotating ring-disk electrodes (RRDE) loading the catalyst was used as the working electrode. Experimentally, 1 mg of the respective catalyst powder was dispersed in 1 mL of ethanol with 50 μL of Nafion solution and ultrasonicated for 15 min. A 20 μL of the resulting solution was dropped onto the glassy-carbon disk (diameter of 3 mm) using a microliter syringe and dried at room temperature. The catalyst loadings were all 0.28 mg cm^−2^. Liner sweep voltammetry (LSV) was performed in nitrogen-statured 0.5 M H_2_SO_4_ at a scan rate of 10 mV s^−1^ at 1400 rpm. Electrochemical impedance spectroscopy (EIS) was measured in the same configuration from 10^−2^ to 10^6^ Hz with modulation amplitude of 5 mV. SCE was calibrated to reversible hydrogen electrode (RHE). A Pt wire was used as the working electrode and the counter electrode, respectively, and the calibration was carried out in a high purity H_2_-saturated electrolyte at a scan rate of 0.1 mV s^−1^. The potential at which the current crossed zero was taken to be the thermodynamic potential for the hydrogen electrode reactions. In 0.5 M H_2_SO_4_, E (RHE) = E (SCE) + 0.26 V. All the potentials reported in our manuscript are against RHE.

## Additional Information

**How to cite this article**: Lu, X. *et al*. One-Step Hydrothermal Fabrication of Three-dimensional MoS_2_ Nanoflower using Polypyrrole as Template for Efficient Hydrogen Evolution Reaction. *Sci. Rep.* **7**, 42309; doi: 10.1038/srep42309 (2017).

**Publisher's note:** Springer Nature remains neutral with regard to jurisdictional claims in published maps and institutional affiliations.

## Supplementary Material

Supporting Information
