# Supplementary material for: One-Step Hydrothermal Fabrication of Three-dimensional MoS2 Nanoflower using Polypyrrole as Template for Efficient Hydrogen Evolution Reaction
Source: Sci Rep. 2017 Feb 14;7:42309. doi: 10.1038/srep42309 (PMC5307311; doi:10.1038/srep42309)
Supplement: Supporting Information [file srep42309-s1.doc]

**Supporting Information**

**One-step hydrothermal fabrication of three-dimensional MoS_2_ Nanoflower using polypyrrole as template for efficient** **hydrogen evolution reaction**

**Xin Lu**^a^**, Yingwu Lin** ^a,b^**,** **Haifeng Dong^b*^, Wenhao Dai^b^, Xin Chen^b^, Xuanhui Qu**^a^**, Xueji Zhang^b*^**

*a Institute for Advanced Materials and Technology, University of Science and Technology Beijing, Beijing 100083, China*

*b Beijing Key Laboratory for Bioengineering and Sensing Technology, ResearchCenter for Bioengineering and Sensing Technology,School of Chemistry & Biological Engineering, University of Science & Technology Beijing, Beijing 100083, P.R. China.*

^*^Corresponding authors.

*E-mail addresses:* [*hfdong@ustb.edu.cn*](mailto:hfdong@ustb.edu.cn) *(H. F. Dong)*; *zhangxueji@ustb.edu.cn (X.J. Zhang)*


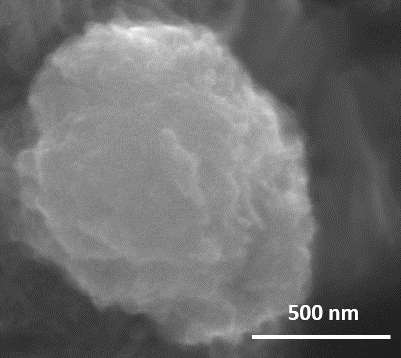


**Fig. S1.** SEM image of PPy.


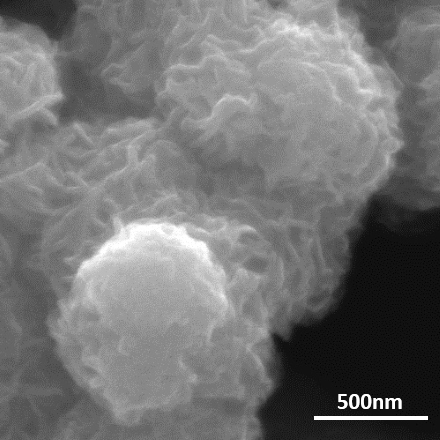


**Fig. S2.** SEM image of MoS_2_-PPy (before annealing).


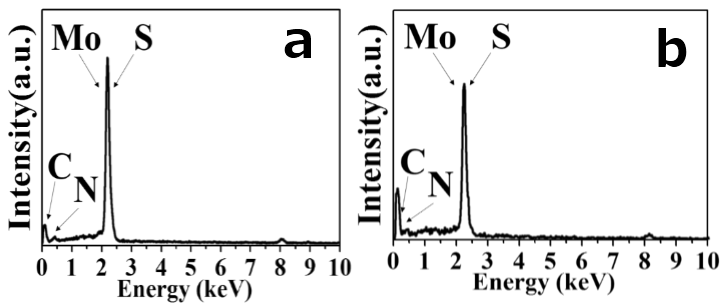


**Fig. S3.** EDS spectrum of MoS_2_-P (a) before annealing and (b) after annealing

**Fig. S4.** XRD spectrum of PPy-MoS_2_ (before annealing).

**Fig. S5.** Thermogravimetric analysis curve of MoS_2_-PPy measured from 50 to

800 ^o^C in air atmosphere with a heating rate of 10 ^o^C/min.


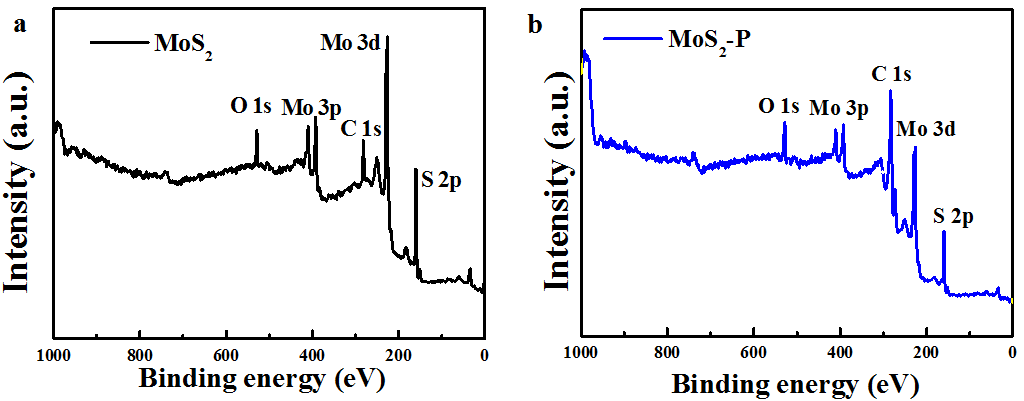


**Fig. S6.** The survey XPS spectrum of (a) MoS_2_ and (b) MoS_2_-P


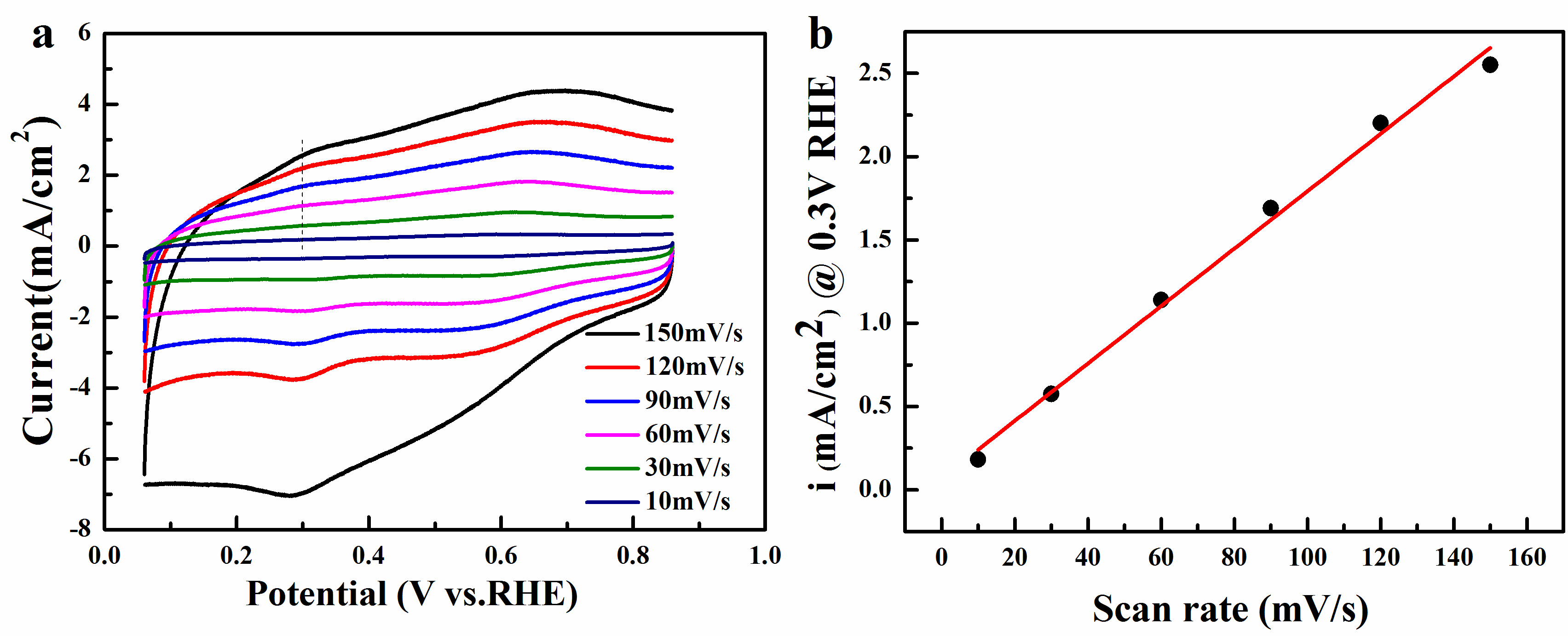


**Fig. S7.** Electrochemical measurement for determining TOF: (a) a cyclic voltammetry (CV) curve of MoS_2_-P at different scan rates. (b) Current density of CV experiment at overpotential +0.3 V vs RHE as a function of scan rates. The slope of this line shows double layer capacitor for MoS_2_-P catalyst system.

**Fig. S8.** (a) The UV−vis spectrum of MoS_2_ and MoS_2_-P. (b) Bandgap determination using Kubelka-Munk Function ([F(R)hv]^1/2^) versus hv plots for MoS_2_-P.

**Table S1.** HER activities of MoS_2_−based catalysts

| Materials | Onset potential (mV) | Tafel slope (mV) | Number of active sites(cm^-2^) | η(mV) | TOF (s^-1^) |
| --- | --- | --- | --- | --- | --- |
| Bulk MoS_2_ | -200 | 143.3 | 1.24×10^16^ | 400 | 0.54 |
| MoS_2_ | -170 | 95.9 | 2.85×10^17^ | 400 | 0.68 |
| MoS_2_-P | -100 | 80.5 | 3.345×10^17^ | 400 | 0.85 |
| Plasma-engineered MoS_2_ [[[1]](#endnote-2)] | -150 | 108 | 7.74×10^16^ | 350 | 1.32 |
| Hierarchical MoS_2_ [[[2]](#endnote-3)] | -50 | 70 |  | 150 | 0.41 |
| Defect-rich MoS_2_ [[[3]](#endnote-4)] | -120 | 50 |  | 300 | 0.72 |
| Irradiated Au−MoS_2_[[[4]](#endnote-5)] | -160 | 71 |  | 300 | 8.76 |
| Double−gyroid MoS_2_ [[[5]](#endnote-6)] | -110 | 50 |  | 200 | 1 |
| Strained vacancies MoS_2_ [[[6]](#endnote-7)] | -30 | 60 | 2.20×10^14^ | 125 | 10 |

1. [?] L. Tao, X. Duan, C. Wang, X. Duan, S. Wang, Chem. Commun. 51 (2015) 7470-7473. [↑](#endnote-ref-2)
2. [?] J. Zhang, S. Liu, H. Liang, R. Dong, X. Feng, Adv. Mater. 27 (2015) 7426-7431. [↑](#endnote-ref-3)
3. [?] J. Xie, H. Zhang, S. Li, R. Wang, X. Sun, M. Zhou, J. Zhou, X.W. Lou, Y. Xie, Adv. Mater. 25 (2013) 5807-5813. [↑](#endnote-ref-4)
4. [?] Y. Shi, J. Wang, C. Wang, T. Zhai, W. Bao, J. Xu, X. Xia, H. Chen, J. Am. Chem. Soc., 137 (2015) 7365-7370. [↑](#endnote-ref-5)
5. [?] K. Jakob, C. Zhebo, B.N. Reinecke, T.F. Jaramillo, Nat. Mater. 11 (2012) 963-969. [↑](#endnote-ref-6)
6. [?] H. Li, C. Tsai, A.L. Koh, L. Cai, A.W. Contryman, A.H. Fragapane, J. Zhao, H.S. Han, H.C. Manoharan, F. Abildpedersen, Nat. Mater. (2015) 1-7. [↑](#endnote-ref-7)
